# Variability of serum reproductive hormones in cows presenting various reproductive conditions in semi-arid areas of the North West Province, South Africa

**DOI:** 10.14202/vetworld.2020.502-507

**Published:** 2020-03-17

**Authors:** K. Molefe, M. Mwanza

**Affiliations:** Department of Animal Health, Faculty of Natural and Agricultural Sciences, Mafikeng Campus, North-West University, Private Bag X 2046 Mmabatho 2735, South Africa

**Keywords:** cows, hormones, reproductive conditions

## Abstract

**Background and Aim:**

Hormones play a significant role in supporting reproductive processes. Predisposition to metabolic disorders may result from biological alterations in the neurohormonal system, thus leading to impaired immune function and poor reproductive performance. The aim of this study was to determine the reproductive hormonal profile in cows with reproductive conditions in semi-arid areas of the North West Province, South Africa, to establish possible correlations between different conditions and the hormonal profile.

**Materials and Methods:**

Blood samples were collected from cows in different communal areas of Mafikeng. Convenience sampling was used to collect samples for the study. Blood samples were collected cows experiencing dystocia (n=50), retained placenta (n=13), downer cow syndrome (n=34), vaginal prolapse (n=16), and abortions (n=69), following cases reported at the Animal Health Hospital of the North-West University, Mafikeng Campus. Descriptive statistics, such as mean and standard deviations, were used to describe the distribution of hormone levels across reproductive conditions. p-value less than the significance level was set at 5% (p<0.05).

**Results:**

Data obtained revealed significantly higher estradiol in abortion (1122.99±71.99 pg/ml), downer cow syndrome (781.32±135.7 pg/ml), and dystocia (862.09±123.44 pg/ml). Oxytocin (OT) differed significantly in cows with dystocia (370.50±71.66 pg/ml) and abortion (574.73±60.65 pg/ml). Significantly low progesterone (Pg) was observed in abortion (2.45±1.509 ng/ml) and dystocia (8.59±0.402 ng/ml) while increased prostaglandin alpha was observed in cows with vaginal prolapse and abortion.

**Conclusion:**

The findings highlight an increase in serum estradiol and OT in aborting cows. Low Pg and estradiol in cows with vaginal prolapses and retained placenta were noted. An association was seen between downer cow syndrome and high concentrations of estradiol and Pg. Prostaglandin alpha may increase in cases of vaginal prolapse and abortion. Hormonal alterations were observed and may contribute to the incidences of different reproductive conditions.

## Introduction

Progesterone (Pg), estrogen, oxytocin (OT), and prostaglandins have both direct and indirect effects on the contraction of myometrium in cows [1]. The pituitary release of OT is influenced by ovarian estrogen and is inhibited by Pg [2]. Strong utero-muscular contractions may occur through the regulation of OT receptors in myometrium triggering prostaglandin F2 alpha (PGF2α) secretion through an increase in estrogen [3,4]. Myometrial contractions initiated by prostaglandins, lead to corpus luteal lysis, which secrete relaxin and decrease in Pg concentrations [4].

Pg is a steroid hormone primarily involved in receptor coordination of female reproductive processes such as pregnancy initiation and maintenance [5]. Pg is produced by the ovaries before pregnancy and by the placenta in the course of pregnancy during which its concentration is elevated [6]. The functional properties of Pg include the preparation of the uterus for pregnancy and maintenance of pregnancy [7]. There is a significant alteration in Pg concentration during abortions in cows [8]. According to Purohit *et al*. [9], the levels of Pg (ng/ml) during gestation range from low (6-15 ng/ml) to high (20-50 ng/ml). Increased Pg due to stress stimulates immunosuppressive protein accumulation in the lumen of the uterus, causing susceptibility to persistent bacterial infections [4,10,11]. An increase in serum Pg and lowered estradiol are seen in cows with retained placental membranes [10]. Changes in the Pg profile and less estrone sulfate production have been linked to the incidence of dystocia (calving difficulty) in cows [8].

Estrogen is an intraovarian reproductive hormone characteristically known to affect calcium homeostasis, the skeleton, liver, hypothalamus, pituitary gland and, influences growth, development, maturation, and sexual differentiation in animals [12]. There are three forms of estrogen as follows: Estrone (E_1_), estradiol (E_2_), and estriol (E_3_). Out of these three forms, estradiol is produced by the ovaries while estriol is secreted by the placenta [13]. The production of estradiol (E_2_) and PGF2α are lowered in cases of placental membrane retention [4]. Elevation in gene expression during parturition is stimulated by estrogen, which contracts and excites the myometrium, as well as increasing OT sensitivity in the uterus throughout the period of gestation [4]. A study by Opara *et al*. [14] revealed that the reference values for estradiol (pg/ml) during pregnancy are 115.7-858.5 pg/ml.

A nonapeptide hormone, OT, is functionally recognized for its ability to regulate reproductive processes such as pregnancy, milk let down and stimulating the influx of calcium in cows [15]. Other functions of OT include contraction of the uterus during labor, stimulation of the uterine, muscle contraction, and also aiding in the increase of prostaglandin production, which also elevates contractions [16]. OT is inhibited by Pg during pregnancy [1]. It is also known to stimulate the synthesis of prostaglandins in the tissue endometrium [17]. The monitoring of OT is done during the peripartum period and is beneficial as a preventive and corrective m1easure for reproductive disorders. Chen *et al*. [2] found that the level of OT (pg/ml) ranges from 8.7 to 51.2 pg/ml during pregnancy.

Prostaglandins (PGF2α) are physiological lipid compounds with hormone-like effects in animals and their functional properties include the regulation of smooth muscle tissues [18]. PGF2α is uterotonic mediators involved in increasing uterine contractility and can as well be used for the treatment of metabolic disorders such as retained placenta [1]. Other functions of prostaglandins include labor induction, estrus synchronization, and stimulation of the production of luteinizing hormone [18]. An increase in prostaglandin has been associated with the occurrence of abortions in cows [8]. The concentration of prostaglandin (pg/ml) in cows during pregnancy ranges from 0.3 to 6.4 ng/ml [19].

The gestation period in cows induces oxidative stress, resulting in immune dysfunctions and high incidences of peripartum disorders [4,20]. Abortions, vaginal prolapse, dystocia, and retained placenta are among some of the main reproductive disorders encountered [21]. Normal mechanical contraction of the uterus depends on the release of prostaglandin and OT hormones at the onset of labor necessary for fetal delivery [4]. In preparation for a new pregnancy, the cow has to return to the non-pregnant state in the postpartum period [22]. However, uterine involution may occur due to several post-parturient reproductive conditions, such as abortion, retained placenta, uterine prolapse, dystocia, subclinical/clinical endometritis, and pyometra, which delay preparation for the subsequent prenatal period and, consequently, decreasing conception rate [23].

The aim of this study was to determine the hormonal profile in cows with reproductive conditions. The role of hormones in cows requires careful consideration before and during pregnancy, as any imbalance can negatively impact its overall reproductive health. Any excess or deficiency of reproductive hormones during the peripartum period produces harmful effects on the health of both the cow and the calf. Hormones are also essential and require much consideration in cows during the production stage.

## Materials and Methods

### Ethical approval

The study was approved following consideration by the Animal Research Ethics Committee after being reviewed by the North-West University Research Ethics Regulatory Committee (NWU-RERC), ethics number: NWU-00409-18-A5.

### Study area and study period

This study was conducted in Mafikeng, North West Province, South Africa from August 2016 to November 2018. The area is geographically located at 25°38‘E and 25°51‘S coordinates. The area has a temperature that ranges from 22 to 35°C, typically experienced during summer (between August and March) with an annual average rainfall of 200-500 mm [24]. During winter, the nights are chilly and days sunny (from May to July) with temperatures ranging from 2 to 20°C [25]. The total surface area of Mafikeng is estimated to be about 3703 km^2^.

The area is divided into 28 wards and 102 suburbs and villages [26]. Mafikeng is located in Ngaka Modiri Molema District and agriculture is the major driving force of the economy, producing mainly cattle and crops.

### Animals, sampling, and processing of samples

The study was conducted using cows from different communal farms in Mafikeng Local Municipality. Blood samples were conveniently collected when reproductive cases were reported to the Animal Health Hospital of the North-West University. A total of 182 blood samples were collected from cows with dystocia (n=50), retained placenta (n=13), downer cow syndrome (n=34), vaginal prolapse (n=16), and abortions (n=69). Samples were collected through venipuncture, using double-pointed Vacutainer 18 gauge needles into red-capped tubes without anticoagulant, then placed on ice in a cooler box and taken for analysis at the laboratory of the North-West University. Serum was harvested by centrifuging blood for 15 min at 1000×*g* at 2~8°C. The serum was then frozen at −20°C pending examination [27].

### Tests

#### Estradiol analysis

Serum was analyzed using the E_2_ (Estradiol) ELISA Kit product of Elabscience, USA (Catalog No: E-EL-0065), in accordance with the manufacturer’s instructions. Briefly, sample preparation and assay were as follows: Samples were allowed to clot for 2 h at room temperature or overnight at 4°C before centrifugation for 15 min at 1000×*g* at 2~8°C. The supernatant was collected to perform the assay. Blood collection tubes should be disposable and non-endotoxin. Some 50 μL of standard or sample was added to each well of the ELISA plate. About 50 μL of horseradish peroxidase (HRP)-labeled estradiol and 50 μl of detection Ab were added to each well. The tubes were incubated for 1 h at 37°C, then aspirated and washed 3 times. About 50 μL of substrate A and substrate B were added to each well and incubated for about 15 min at 37°C, with shading light. About 50 μL stop solution was also added to the mixture. The sample was read at 450 nm immediately using a microplate reader and the results calculated.

#### Analysis of Pg

Blood samples were collected into red stoppered tubes, clotted at 4°C for 24 h, and centrifuged for 15 min at 2500×*g* for the collection of serum. Serum samples were stored at −20°C until assayed for Pg concentrations. Serum Pg concentrations were analyzed using Pg ELISA (enzyme immunoassay) Kit (Catalog No: E-EL-0065) obtained from Elabscience, USA, as previously described [28].

#### Analysis of prostaglandins

The samples were analyzed using the Human PGF2α (Prostaglandin) ELISA Kit (Catalog No: E-EL-H1841) in accordance with the manufacturer’s instructions (Elabscience^®^, USA**)**. As part of the procedure, blood samples were allowed to clot for 2 h at room temperature or overnight at 4°C before centrifugation for 15 min at 1000×*g* at 2~8°C. The supernatant was collected to perform the assay. Briefly, about 50 μL of standard or sample was added to each well of the ELISA plate and 50 μL of biotinylated detection Ab also added to each well. The plate was incubated for 45 min at 37°C. The supernatant was aspirated and washed 3 times. Some 100 μL HRP conjugate was also added to each well. The plate was incubated for 30 min at 37°C. The supernatant was aspirated and washed 5 times. About 90 μL of substrate reagent was added to the mixture. The mixture was incubated for 15 min at 37°C. Thereafter, 50 μL stop solution was added to the mixture. After a reading was done at 450 nm, the results were calculated using a microplate reader set at 450 nm.

#### Analysis of OT

Samples were examined using the OT ELISA Kit (Catalog No: E-EL-0029) product of Elabscience^®^ (USA). As part of the procedure, blood samples were allowed to clot for 2 h at room temperature or overnight at 4°C before centrifugation for 15 min at 1000×*g* at 2~8°C. The supernatant was collected to perform the assay. Briefly, about 50 μL of standard or sample was added to each well of the ELISA plate and 50 μL of biotinylated detection Ab also added to each well. The plate was incubated for 45 min at 37°C. The supernatant was aspirated and washed 3 times. Some 100 μL HRP conjugate was added to each well. The plate was incubated for 30 min at 37°C. The supernatant was aspirated and washed 5 times. About 90 μL of substrate reagent was added. The preparation was incubated for 15 min at 37°C. Thereafter, 50 μL stop solution was added. After a reading was done at 450 nm, the results were calculated using a microplate reader set at 450 nm.

### Statistical analysis

In this study, sample readings were recorded in Microsoft Excel (2010). Data were analyzed using the Statistical Package for the Social Sciences (SPSS) version 25. Descriptive statistics such as mean, standard deviation, and skewness were used to describe the distribution of the hormone levels across reproductive conditions. Multivariate analysis of variance (MANOVA) was used to compare the hormone levels across reproductive conditions. MANOVA was also useful in determining if there was a significant difference in the hormone levels according to the different reproductive conditions. The test statistic (F) for Wilk’s MANOVA test was used. A significant p-value of the F-statistic of the unbalance factorial design (p-value less than the significance level of 5%) indicates that the independent variable has an impact on the level of hormones.

## Results

Table-1 shows that the mean and standard deviations of the levels of hormones were different across the reproductive conditions. The results also show that the mean levels of Pg, estradiol, prostaglandin, and OT were significantly (p<0.05) different in the reproductive conditions (Table-1).

**Table-1:** Mean±standard deviation of hormones in cows with reproductive conditions.

Reproductive conditions	Hormones		

	Estradiol (pg/ml)	Prostaglandin (pg/ml)	Oxytocin (pg/ml)
Abortion	1122.99±71.99*	300.41±24.48**	574.73±60.65*
Dystocia	862.09±123.44*	153.06±43.61^ns^	370.50±71.66*
Vaginal prolapse	187.94±91.44*	241.84±28.35**	433.53±14.07^ns^
Downer cow syndrome	781.32±135.70*	205.13±25.08**	602.73±0.15^ns^
Retained placenta	312.39±112.37*	92.06±45.57**	598.66±0.10^ns^

(*)=p<0.001, (**)=p<0.05, NS=Not significant, Pg/ml=Picogram/milliliter

In Table-2, the results show that the MANOVA test was significant at 5% (p<0.05), which suggests that the mean levels of hormones differ across the various reproductive conditions. Table-3 shows that the mean levels of Pg, estradiol, prostaglandin, and OT differ significantly across the reproductive conditions (p<0.05).

**Table-2:** MANOVA test used to estimate the significance of the relationship between reproductive conditions and hormones (estradiol, progesterone, prostaglandin, and oxytocin).

Multivariate tests	Value	F	Hypothesis df	Error df	Sig.
Pillai’s trace	0.767	9.966	16.000	672.000	0.000
Wilks’ lambda	0.358	12.613	16.000	504.721	0.000
Hotelling’s trace	1.462	14.938	16.000	654.000	0.000

**Table-3:** Tests of between-subjects effects showing significant values of hormones in reproductive conditions (abortion, retained placenta, vaginal prolapse, dystocia, and downer cow syndrome).

Source	Type III sum of squares	df	Mean square	F	Sig.
Reproductive condition					
Progesterone (ng/ml)	2034.520	4	508.630	8.145	0.000
Estradiol (pg/ml)	16,347,109.821	4	4,086,777.455	17.968	0.000
Prostaglandin (pg/ml)	1,414,698.459	4	353,674.615	24.654	0.000
Oxytocin (pg/ml)	1,505,616.169	4	376,404.042	3.693	0.007

**Figure-1:**
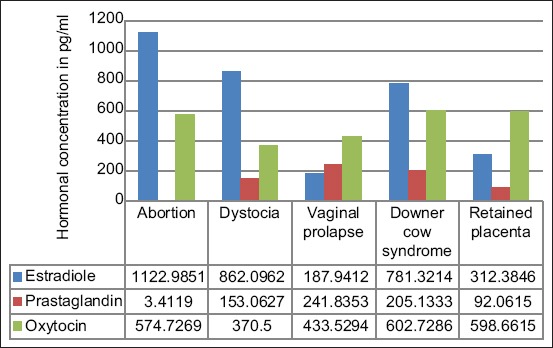
Concentrations of estradiol, prostaglandins, and oxytocin serum in cows with different reproductive conditions.

The highest concentrations of estradiol were found in cases of abortion followed by dystocia and downer cow syndrome while the least concentration was observed in cases of vaginal prolapse (Figure-1). Prostaglandin was higher in vaginal prolapses while the least was in the retained placenta. OT concentrations were equally high in retained placenta and downer cow syndrome (Figure-1).

**Figure-2:**
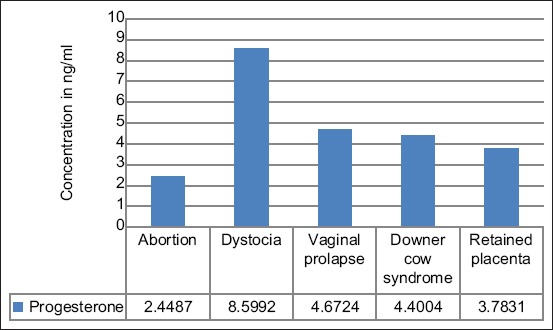
Concentration of progesterone serum in cows with different reproductive conditions.

The concentration of Pg was highest in cases of dystocia, followed by vaginal prolapse, downer cow syndrome, and retained placenta while the least was observed in aborting cows (Figure-2). However, the mean concentrations of Pg (ng/ml) were within the normal range in cases of dystocia (Table-4).

**Table-4:** Mean±standard error of progesterone in different reproductive conditions.

Reproductive conditions	Progesterone (ng/ml)
Abortion	2.45±1.509*
Dystocia	8.59±0.402
Vaginal prolapse	4.67±0.301*
Downer cow syndrome	4.40±1.222*
Retained placenta	3.78±0.151*

* p<0.01, ng/mg=Nanogram/milliliter

## Discussion

The regulation of prostaglandin (PGF2α) secretion in ruminants is through estradiol (E_2_), Pg, and OT [19]. Through a positive feedback loop, PGF2α’s primary release triggers corpus luteal production of OT and E_2_ increases PGF2α secretion through epithelial cell OT stimulation in the uterus of cows [19]. The results revealed significantly (p<0.001) different hormonal levels in cows suffering from reproductive conditions (Table-2).

The mean estradiol (pg/ml) concentrations significantly increased in cases of abortion (1122.99±71.99), downer cow syndrome (781.32±135.7), and dystocia (862.09±123.44) (Table-1 and Figure-1). The results obtained in this study are similar to those of the previous studies, which revealed that significantly higher levels of circulating estrogen are typically seen during gestation [13]. Adversely, a study by Mehrvar *et al*. [29] revealed that incidences of calf mortality were due to a relaxation deficiency of the pelvic ligaments due to insufficient estradiol.

The lowest concentrations of estradiol were observed in cases of vaginal prolapse (187.94±91.44) and retained placenta (312.39±112.37), as shown in Table-1 and Figure-1. A deficiency of estrogen, which is responsible for the movements of connective tissues, such as relaxation and contractions, predisposes cows to vaginal prolapse [30]. The results are also in line with a study that revealed a relationship between lowered E_2_ and vaginal prolapse in cows [9]. On the contrary, estradiol values obtained in the present study were above those reported for pregnant cows in a study by Opara *et al*. [14]. This could have resulted from differences in the health status of cows compared to the current study.

Prostaglandins (PGF2α) are uterotonic mediators responsible for the increase in muscle contractility [1]. The current study showed that the mean PGF2α (pg/ml) values were significantly higher in cases of vaginal prolapse (241.84±28.35) and abortion (300.41±24.48), as shown in Table-1. Prostaglandins released due to protozoal invasion of the placenta and the heart can cause abortion in cows, whereby embryo tissue is destroyed due to the insufficiency of oxygen and lack of nutrients [31]. Vaginal prolapses may concurrently be seen as a result of abortions in cows [32]. This could explain the observed increase of prostaglandin levels in both conditions.

Moreover, changes in luteal blood causing luteal lysis induced by prostaglandins are usually followed by lowered production of Pg, which then leads to abortion [19]. Several other studies have revealed negative effects (embryo mortalities, deformities, and stillbirths) due to elevated prostaglandin alpha (PGF2α) concentrations on the survival of the embryo [19,33-35].

OT is a paraventricular and supraoptic nuclei pituitary secreted hormone known to positively influence uterine contractility, uterine involution, uterine motility, calving, the expulsion of uterine contaminants, and stimulation of prostaglandins (PGF2) synthesis [36]. OT (pg/ml) concentrations differed significantly, higher in cows with dystocia (370.50±71.66) and abortion (574.73±60.65), as shown in Table-1 and Figure-1. The values were higher than the mean concentrations obtained in a study by Chen *et al*. [2], which revealed an OT range of 8.7-51.2 pg/ml in pregnant cows.

Another observation in the present study was that OT levels were high in incidences of vaginal prolapse (433.53±14.07), retained placenta (598.66±0.10), and downer cow syndrome (602.73±0.15) but not significantly different (Table-1). There were myometrial contractility upsurges due to the production of OT [9]. OT stimulation of milk let down could explain the high values of OT in cows with downer cow syndrome, which may occur due to loss of calcium ions through the production of milk, thus causing cows to be a downer.

Low concentrations of Pg in cows oscillated between 6 and 15 ng/mL throughout pregnancy, whereas the highest mean values ranged from 20 to 50 ng/ml throughout gestation [9]. A high concentration of Pg was sustained during gestation and was the best biomarker for the evaluation of reproductive status [37]. The study revealed significantly low concentrations of Pg (ng/ml) mean in cows with abortion (2.45±1.509), dystocia (8.59±0.402), vaginal prolapse (4.67±0.301), downer cow syndrome (4.40±1.222), and retained placenta (3.78±0.151) (Table-4 and Figure-1). Low Pg level has been linked to fetal mortalities due to its relations with poor nutrition, thus resulting in negative energy balance, which, therefore, interferes with the microenvironment in the uterus, causing an abortion in cows [19]. Any hormonal imbalance in cows can negatively affect productivity through cow/calf losses and impaired reproduction health with both direct and indirect effects.

## Conclusion

The levels of estradiol and OT are likely to increase in aborting cows while lowering Pg. Vagina prolapse and retained placenta in cows are linked to reduced levels of estradiol and Pg. High estradiol and OT and concentrations are related to dystocia. Cases of downer cow syndrome presented high serum Pg and estradiol levels. Prostaglandin alpha may increase in cases of vaginal prolapse and abortion. Hormonal alterations were observed and may contribute to different reproductive conditions.

## Authors’ Contributions

MK collected the samples and performed the statistical analysis of the data. MM conceived the idea and supervised the study. Both authors conducted laboratory tests, wrote and revised the manuscript, and approved the final manuscript for submission.
